# Supplementary figures and images for: Mass-Spectrometry-Based Lipidomics Discriminates Specific Changes in Lipid Classes in Healthy and Dyslipidemic Adults
Source: Metabolites. 2023 Feb 3;13(2):222. doi: 10.3390/metabo13020222 (PMC9964724; doi:10.3390/metabo13020222)

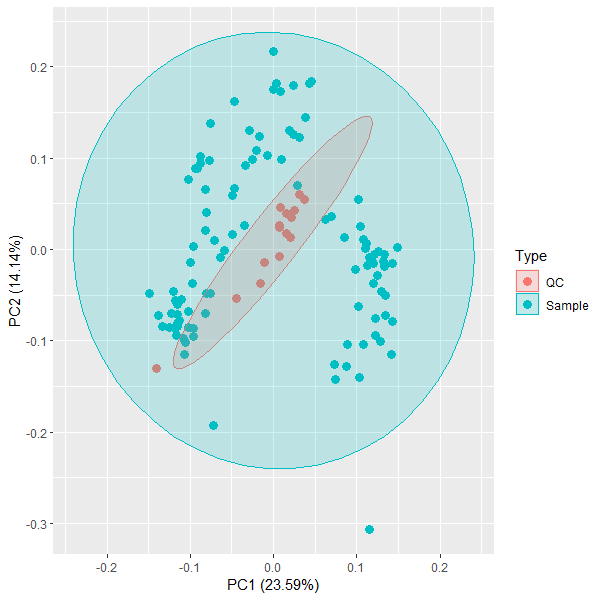

Supplement: Supplementary file 1 [file metabolites-13-00222-s001.zip › Additional file 5.png]

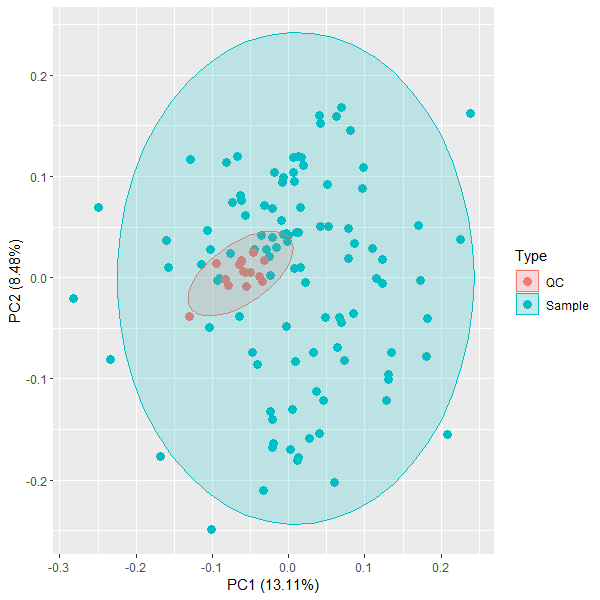

Supplement: Supplementary file 1 [file metabolites-13-00222-s001.zip › Additional file 4.png]
